# Interactive effect of phenylalanine with duration of diabetes on the risk of small vessel disease in Chinese patients with T2DM

**DOI:** 10.3389/fendo.2024.1472967

**Published:** 2025-01-03

**Authors:** Jun Zheng, Wei Zhang, Yu-Yang Miao, Xue-Rui Li, Wei-Ming Luo, Xi-Lin Yang, Zhong-Ze Fang, Qiang Zhang

**Affiliations:** ^1^ Department of Geriatrics, Tianjin Medical University General Hospital, Key Laboratory of Post-Neuroinjury Neuro-Repair and Regeneration in the Central Nervous System, Ministry of Education, State Key Laboratory of Experimental Hematology, Tianjin Key Laboratory of Elderly Health, Tianjin Geriatrics Institute, Tianjin, China; ^2^ Department of Epidemiology and Biostatistics, School of Public Health, Tianjin Medical University, Tianjin, China; ^3^ Department of Toxicology and Sanitary Chemistry, School of Public Health, Tianjin Medical University, Tianjin, China

**Keywords:** type 2 diabetes mellitus, small vessel disease, phenylalanine, duration of diabetes, T2DM

## Abstract

**Aims:**

Few prior studies have explored the relationship between phenylalanine and diabetic small vessel disease (SVD) in patients with different durations of type 2 diabetes mellitus(T2DM). Our study aimed to explore whether phenylalanine is associated with the risk of SVD and to further explore whether phenylalanine interacted with the duration of T2DM to alter the risk of SVD.

**Materials and methods:**

A total of 1,032 T2DM patients were enrolled using the Liaoning Medical University First Affiliated Hospital (LMUFAH) system. SVD was defined as patients with diabetic nephropathy (DN) or diabetic retinopathy (DR) alone, or both. Serum amino acids were measured by mass spectrometry (MS) technology. A binary logistic regression model was used to examine associations of phenylalanine with SVD risk. Restricted cubic spline (RCS) regression was used to draw the odds ratio curves of plasma phenylalanine for SVD. Additive interaction analysis was employed to test the interaction of low phenylalanine with a long duration of T2DM for SVD.

**Results:**

Among the 1,032 T2DM patients, 286 (27.7%) had SVD. Phenylalanine <42μmol/L was associated with a markedly increased risk of SVD (OR 1.76, 95%CI 1.23 to 2.51), which was enhanced by a duration of T2DM of ≥5 years to 4.83 (95%CI 2.97-7.87) with significant additive interactions. The inclusion of phenylalanine and duration of T2DM into a traditional risk factor model substantially increased the area under the receiver operating characteristic curve from 0.67 to 0.71 (95% CI 0.70 to 0.75) (P <0.05).

**Conclusions:**

In Chinese patients with T2DM, phenylalanine <42μmol/L was associated with an increased risk of SVD, which was further amplified by a duration of T2DM of ≥5 years.

## 1 Introduction

The prevalence of type 2 diabetes mellitus (T2DM) and its complications has resulted in an enormous burden of death and disability worldwide. Complications from T2DM are very common, with half of the patients with T2DM in an observational study of 28 countries in Asia, Africa, South America, and Europe presenting with small vessel disease (SVD) and 27% with macrovascular diseases (1). It is therefore clear that SVD is more prevalent than macrovascular diseases. Diabetic vascular complicating diseases are the primary cause of mortality in diabetes sufferers, the most common of which are diabetic nephropathy (DR) and diabetic retinopathy (DN) (2). According to the epidemiology survey of T2DM in large Chinese cities, DR and DN account for 39.7% and 31.5% of microangiopathy in diabetes, respectively (2). Furthermore, a comparatively low frequency of macrovascular disease in Chinese individuals with diabetes was confirmed in the follow-up study of incident complications (3).

Although standardized treatments based on the strict control of blood glucose, blood pressure, and blood lipids have been verified to be able to slow the progression of diabetic microangiopathy, they have not completely blocked or reversed the disease (2, 4, 5). Hence, expanding the current knowledge on the physiopathology of SVD and identifying novel potential biomarkers may help to facilitate the detection and management of SVD.

In recent years, amino acids and related metabolites in the blood have been intensely studied as some of the most promising biomarker candidates for T2DM and its complications. It is worth noting that a substantial number of studies have shown that circulating amino acid levels are altered in patients with T2DM as well as SVD. Aromatic amino acids (AAAs), i.e., tyrosine, phenylalanine, and tryptophan, play an important role in energy metabolism as substrates of oxaloacetate (6, 7), which can indirectly promote gluconeogenesis and affect the secretion of insulin and glucagon (8). A 6-year-long cohort study found that AAAs were predictors of insulin resistance (9). In cross-sectional studies, phenylalanine shows the most consistent positive associations with prediabetes and insulin resistance (10, 11).

Currently, the association of phenylalanine with SVD in T2DM patients remains controversial. A case-cohort study involving 11,140 participants with T2DM found no prospective association between phenylalanine and SVD (12). Nevertheless, some studies have also found that phenylalanine may be related to SVD to a certain extent by regulating vascular endothelial function and the production of neurotransmitters (13, 14). Furthermore, dysregulation of phenylalanine and impaired conversion to tyrosine (15) may also contribute to the pathogenesis of SVD (16). Beyond this, whether the duration of diabetes affects the association between phenylalanine and SVD in T2DM is currently unknown.

Therefore, we conducted a retrospective study to explore the association between phenylalanine and SVD in T2DM and further tested whether there is an interaction between phenylalanine and the duration of diabetes on the risk of SVD.

## 2 Materials and methods

### 2.1 Study design and population

We used a retrospective study design to evaluate the association between phenylalanine and small vessel disease in T2DM. We retrieved electronic medical records of 1,898 patients with a diagnosis of T2DM at the Liaoning Medical University First Affiliated Hospital (LMUFAH), Jinzhou, China who were admitted to the hospital from May 2015 to August 2016 (17). The inclusion criteria were as follows: age ≥18 years; complete information for height, weight, and blood pressure was available. Based on these criteria, 1,032 patients with T2DM who were diagnosed according to the 1999 World Health Organization’s criteria (18) or who were treated with antidiabetic drugs remained in our current analysis. The patients were divided into two groups: SVD and non-SVD. The LMUFAH Clinical Research Ethics Committee approved the study, and informed consent was waived due to the retrospective character of the study, which is consistent with the Helsinki Declaration.

### 2.2 Data collection

We retrospectively extracted clinical information from the electronic medical system (EMS), including demographics, anthropometrics, biochemical test parameters, medications, and disease status. Age was automatically determined by the EMS as the gap between admission year and birth year. Body weight, height, and blood pressure were measured by specially trained doctors and nurses using standardized methods. Body mass index (BMI) was calculated as weight/height^2^ (kg/m^2^). Venous blood samples were taken in the morning after fasting for at least 8 hours. Lipids, i.e., high-density lipoprotein cholesterol (HDL-C), low-density lipoprotein cholesterol (LDL-C), triglyceride (TG), and glycated hemoglobin (HbA1c) were examined in the central laboratory of the hospital. Disease status included duration of diabetes, DR, DN, coronary heart disease (CHD), and stroke.

Use of oral anti-diabetic drugs (OAD), insulin, angiotensin-converting enzyme inhibitors (ACEIs), angiotensin receptor blockers (ARBs), and beta-blockers, statins, and other lipid-lowering drugs in hospital was documented.

### 2.3 Clinical definitions

BMI was classified into four categories: underweight (<18.5 kg/m^2^), normal weight (18.5-23.9 kg/m^2^), overweight (24-27.9 kg/m^2^), and obese (≥28 kg/m^2^) as recommended by the National Health Commission in China (19). According to the criteria recommended by the American Diabetes Association, the reasonable HbA1c goal was<7.0% (20). We divided the duration of diabetes into two categories according to the median (5 years).

Stroke was defined as subarachnoid hemorrhage, cerebral venous thrombosis, spinal cord stroke, or ischemic stroke. CHD was defined as having a history of angina with an abnormal electrocardiogram or on a stress test, myocardial infarction, angina coronary artery bypass graft surgery, or angioplasty. DR was detected by bilateral fundus photography and was diagnosed by clinical manifestations of vascular abnormalities in the retina including microaneurysms, retinal hemorrhages, hard exudates, or vitreous hemorrhage. The clinical diagnosis of DN was defined as persistent albuminuria or progressive reduction in estimated glomerular filtration rate (eGFR) with or without retinopathy and was judged by clinicians (21). In our study, SVD was defined as patients with DN or DR alone, or both (12).

### 2.4 Measurement of amino acids

A previous study described the measurement of amino acids in detail (22). Briefly, the metabolomics approach was based on mass spectrometry (MS) technology. We collected whole blood after at least 8h fasting which was stored as a dried blood spot (DBS) and used in the metabolomic assay. Metabolites in the DBS were measured by direct infusion MS technology equipped with AB Sciex 4000 QTrap system (AB Sciex, Framingham, MA, USA). High-purity water and acetonitrile from Thermo Fisher (Waltham, MA, USA) were used as the diluting agent and mobile phase, respectively. 1-Butanol and acetyl chloride from Sigma-Aldrich (St Louis, MO, USA) were used to derive samples. Isotope-labeled internal standard samples of 12 amino acids (NSK-A) were purchased from Cambridge Isotope Laboratories (Tewksbury, MA, USA) while standard samples of the amino acids were purchased from Chrom systems (Grafelfing, Germany).

### 2.5 Statistical analysis

Normally distributed continuous data were expressed as mean ± standard deviation (SD), while skewed data were expressed as median (interquartile range). A Q-Q plot was used to test the normality. Qualitative variables were reported as frequencies (%). To determine significant differences between the two groups, Student’s t-test or the Mann–Whitney U-test was used for the continuous data, while the Chi-square test (or Fisher’s test when appropriate) was used for categorical variables.

A binary logistic regression model was used to obtain the odds ratio (OR) at a 95% confidence interval (CI) of phenylalanine for the risk of SVD. A structured adjustment scheme was established to adjust for the effect of traditional risk factors on T2DM patients with SVD. We obtained univariable OR values and the ORs after adjusting for age, gender, duration of diabetes, BMI, smoking, drinking alcohol, systolic blood pressure (SBP), TG, HDL-C, HbA1c, tyrosine and the use of anti-diabetes drugs, lipid-lowering drugs, and antihypertensive drugs. Restricted cubic spline (RCS) nested in the logistic regression was used to check potential non-linear associations between amino acid levels with the risk of SVD. An RCS curve is a smoothing curve that was used to provide an intuitive non-linear association between amino acid levels with the risk of SVD. In our analysis, we chose four knots (23). As previously (17), we selected a cut-off point by visually checking the curve where the OR of phenylalanine for SVD started to rise rapidly.

We repeated the logistic regression analysis in groups with different diabetes durations to obtain the OR values. Additive interaction analysis was used to verify the relationship between the duration of diabetes (≥5 or <5 years) and phenylalanine for SVD. We calculated the relative excess risk due to interaction (RERI), attributable proportion due to interaction (AP), and synergy index (S) to estimate additive interactions; RERI>0, AP>0, or S>1 indicated a significant additive interaction (24).

Areas under the receiver operating characteristic curves were compared to provide an estimate of the potential predictive value of phenylalanine and the duration of diabetes beyond the traditional risk factors for SVD.

All analyses were performed using SAS version 9.4 (SAS Institute Inc., Cary, NC, USA) and a two-sided p-value of <0.05 in all results was accepted as statistically significant.

## 3 Results

### 3.1 Characteristics of the study participants

The clinical and biochemical characteristics of the study participants are shown in **Tables 1**, **2**. The mean age of the 1,032 participants was 57.2 (SD 13.8) years, the mean BMI was 25.3 (SD 3.9) kg/m^2^, and the mean duration of T2DM was 5 (0-10) years. Of the 1,032 patients, 53.2% were male and 286 had SVD. There were 210 (20.4%) and 199 (19.3%) individuals diagnosed with CHD and stroke, respectively. Patients with SVD were older, comprised more women, and had a longer duration of diabetes, higher SBP, and greater use of insulin and ACEIs. Furthermore, patients with SVD had lower β-blockers use, and CHD and stroke were less prevalent compared with those without SVD. Phenylalanine was lower in SVD than in non-SVD.

**Table 1:** Clinical characteristics of patients with T2DM divided into groups according to the presence of small vessel disease.

Variables	Total(n=1032)	SVD(n=286)	Non-SVD(n=746)	*P*-value
Age	57.2 ± 13.8	58.7 ± 11.7	56.7 ± 14.5	0.019
Duration of diabetes, years	5(0-10)	10(3-16)	3(0-9)	<0.001
<5	501(48.6%)	81(28.3%)	420(56.3%)	<0.001
≥5	531(51.4%)	205(71.7%)	326(47.3%)	
Male sex	549(53.2%)	138(48.3%)	411(55.1%)	0.048
BMI, kg/m^2^	25.3 ± 3.9	25.5 ± 3.7	25.2 ± 3.9	0.288
BMI < 18.5	27(2.6%)	3(1.1%)	24(3.2%)	0.136
BMI ≥18.5 and < 24	352(34.1%)	106(37.1%)	246(33.0%)	
BMI ≥24 and < 28	430(41.7%)	112(39.2%)	318(42.6%)	
BMI ≥28	223(21.6%)	65(22.7%)	158(21.2%)	
Smoking, yes	331(32.1%)	85(29.7%)	246(33.0%)	0.316
Drinking alcohol, yes	290(228.1%)	79(27.6%)	211(28.3%)	0.832
SBP, mmHg	140.4 ± 23.9	145.7 ± 24.6	138.4 ± 23.4	<0.001
DBP, mmHg	82.5 ± 13.5	82.8 ± 13.5	82.3 ± 13.5	0.629
Antidiabetic agents	864(83.7%)	254(88.8%)	610(81.8%)	0.006
Insulin	772(74.8%)	245(85.7%)	527(70.6%)	<0.001
OAD	562(54.5%)	152(53.2%)	410(55.0%)	0.601
Hypotensive agents	413(40.0%)	145(50.7%)	268(35.9%)	<0.001
ACEI	135(13.1%)	60(21.0%)	75(10.1%)	<0.001
ARB	133(12.9%)	44(15.7%)	89(11.9%)	0.104
β-blockers	93(9.0%)	16(5.6%)	77(10.3%)	0.018
Lipid-lowering agents	388(37.6%)	120(42.0%)	268(36.0%)	0.073
Statins	370(35.9%)	112(39.2%)	258(34.6%)	0.170
Other lipid-lowering agents	23(2.2%)	9(3.2%)	14(1.9%)	0.216
Coronary heart disease	210(20.4%)	51(17.8%)	159(21.3%)	0.214
Stroke	199(19.3%)	45(15.7%)	154(20.6%)	0.074
DR alone	162(15.7%)			
DN alone	188(18.2%)			
Both DN and DR	64(6.2%)			

Data are mean (SD), median (IQR), or n (%). The p-value was acquired by comparing the SVD and non-SVD groups; p-values were derived from independent samples.

Student’s t-test was used for the normally distributed variables, the Mann–Whitney U test for skewed distributions, and the χ2 test (or Fisher’s test, if appropriate) for the categorical variables.

SVD, small vessel disease; SBP, systolic blood pressure; DBP, diastolic blood pressure; T2DM, type 2 diabetes mellitus; OAD, Oral anti-glucose drugs; ACEI, ACE inhibitor; ARB, angiotensin receptor blocker; DR, diabetic retinopathy; DN, diabetic nephropathy.

**Table 2:** Biochemical characteristics of patients with T2DM divided into groups according to the presence of small vessel disease.

Variables	Total(n=1032)	SVD(n=286)	Non-SVD(n=746)	*P*-value
Scr, μmol/L	59.6(49.2-74.3)	60.3(49.0-82.1)	59.5(49.2-72.0)	0.143
HDL-C, mmol/L	1.0 (0.9-1.3)	1.0(0.9-1.3)	1.0(0.8-1.2)	0.038
<1.00 in men or <1.30 in women	494(47.9%)	119(41.6%)	375(50.3%)	0.035
≥1.00 in men or ≥1.30 in women	247(23.9%)	73(25.2%)	174(23.3%)	
Unkown	291(28.2%)	94(32.9%)	197(26.4%)	
LDL-C, mmol/L	2.8 (2.2-3.4)	2.8(2.3-3.5)	2.8(2.1-3.4)	0.378
<2.60	307(29.7%)	76(26.6%)	231(31.0%)	0.099
≥2.60	434(42.1%)	116(40.6%)	318(42.6%)	
Unkown	291(28.2%)	94(32.9%)	197(26.4%)	
Triglyceride, mmol/L	1.7 (1.1-2.4)	1.7(1.1-2.4)	1.6(1.1-2.4)	0.281
<1.70	383(37.1%)	90(31.5%)	293(39.3%)	0.031
≥1.70	361(35.0%)	102(35.7%)	259(24.7%)	
Unkown	288(27.9%)	94(32.8%)	194(39.0%)	
HbA1c, %	9.6 ± 2.4	9.4 ± 2.3	9.7 ± 2.4	0.249
<7	77(7.5%)	26(9.1%)	51(6.8%)	0.073
≥7	554(53.7%)	164(57.3%)	390(52.3%)	
Unkown	411(39.8%)	96(33.6%)	315(40.9%)	
Tyr, μmol/L	45.8(36.7-56.3)	41.9(33.1-51.8)	47.1(38.3-58.0)	<0.001
Phenylalanine, μmol/L	45.3(37.1-55.0)	41.3(34.5-51.0)	46.9(38.6-56.8)	<0.001
<42	411(39.8%)	150(52.5%)	261(35.0%)	<0.001
≥42	621(60.2%)	136(47.5%)	485(65.0%)	

Data are mean (SD), median (IQR), or n (%). The p-value was acquired by comparing the SVD and non-SVD groups; p-values were derived from independent samples.

Student’s t-test was used for the normally distributed variables, the Mann–Whitney U test for skewed distributions, and the χ2 test (or Fisher’s test, if appropriate) for the categorical variables.

SVD, small vessel disease; HbA1c, glycated hemoglobin; Scr, serum creatinine; HDL-C, high-density lipoprotein cholesterol; LDL-C, low-density lipoprotein cholesterol; Tyr, tyrosine.

### 3.2 The relationship between phenylalanine and small vessel disease

**Figure 1:**
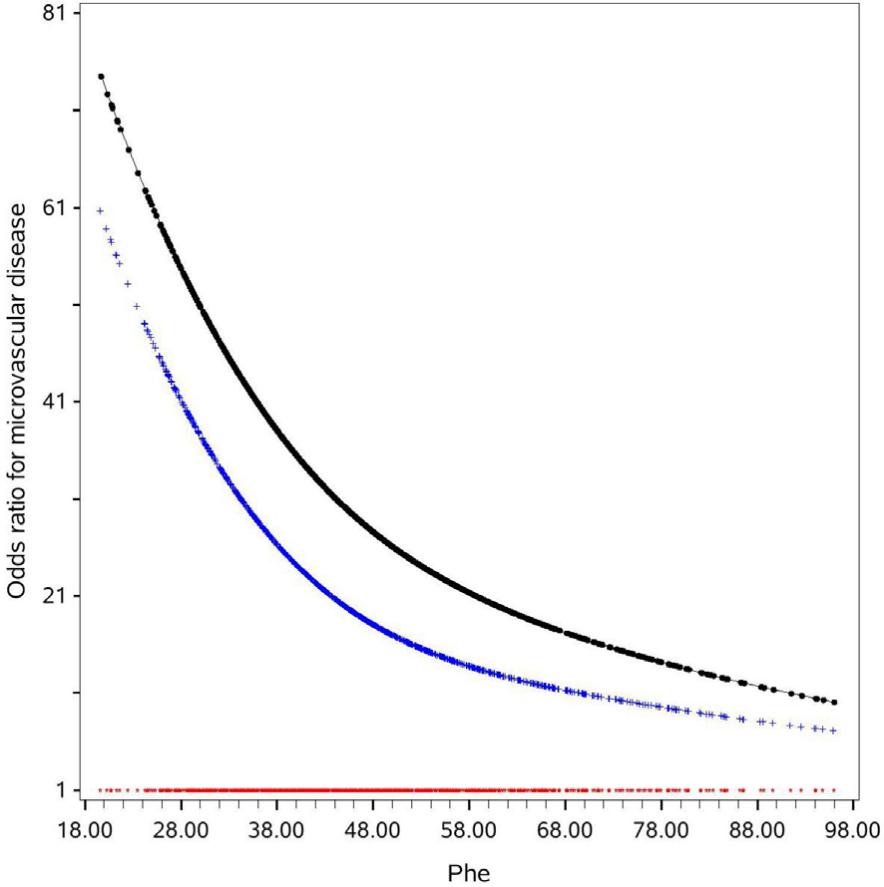
OR curves of phenylalanine for small vessel disease in Chinese patients with T2DM. The black curve was derived from a univariable analysis and the blue curve was derived from a multivariate analysis that adjusted for age, gender, body mass index (<18.5, 18.5-23.9, 24-27.9 and ≥28 kg/m^2^), smoking, drinking alcohol, duration of diabetes, systolic blood pressure, high-density lipoprotein cholesterol (<1.0 mmol/L in men or <1.3 mmol/L in women, ≥1 mmol/L in men or ≥1.3 mmol/L in women, unknown), low-density lipoprotein cholesterol (<2.6 and ≥2.6 mmol/L, unknown), HbA1c (<7%, 7%~8%, and ≥8%, unknown), triglyceride (<1.7 and ≥1.7 mmol/L, unknown), antidiabetic drugs, lipid-lowering drugs, and antihypertensive drugs use. The red curve indicates the reference level (i.e., the OR for small vessel disease was 1). The missing values of HbA1c and lipids were presented as one category. HbA1c, glycated hemoglobin; Phe, phenylalanine; T2DM, type 2 diabetes mellitus.

Plasma phenylalanine concentration and SVD were negatively correlated in a non-linear relationship. A phenylalanine level below 42μmol/L was associated with a markedly increased risk of SVD (**Figure 1**). In a univariable analysis, low levels of plasma phenylalanine were positively associated with the risk of SVD (OR: 2.05, 95% CI: 1.55, 2.70). After adjusting for traditional risk factors, i.e., age, gender, BMI, duration of diabetes, drinking alcohol, smoking, SBP, HDL-C, TG, HbA1c, antidiabetic drugs, lipid-lowering drugs, antihypertensive drugs, and tyrosine, the positive association was strengthened in multivariable model 2 (OR: 1.76, 95% CI: 1.23, 2.51) (**Table 3**).

**Table 3:** Odds ratio of phenylalanine levels for the risk of small vessel disease.

	OR (95% CI)	*P*-value
Univariable model		
Phenylalanine, per μmol/L	0.97 (0.96-0.98)	<0.001
<42 μmol/L	2.05 (1.55-2.70)	<0.001
≥42 μmol/L	reference	
Multivariable model 1		
Phenylalanine, per μmol/L	0.97 (0.96-0.98)	<0.001
<42μmol/L	2.20(1.61-3.01))	<0.001
≥42 μmol/L	reference	
Multivariable model 2		
Phenylalanine, per μmol/L	0.98(0.96-0.99)	0.002
<42μmol/L	1.76(1.23-2.51)	0.002
≥42 μmol/L	reference	

Model 1 was adjusted for age, gender, body mass index (<18.5, 18.5-23.9, 24-27.9 and ≥28 kg/m^2^), duration of diabetes, smoking, drinking alcohol, systolic blood pressure, high-density lipoprotein cholesterol (<1.0 mmol/L in men or <1.3 mmol/L in women, ≥1 mmol/L in men or ≥1.3 mmol/L in women, unknown), triglyceride (<1.7 and ≥1.7 mmol/L, unknown), antidiabetic drugs, lipid-lowering drugs, and antihypertensive drugs use. Model 2 was adjusted for the variables in model 1 plus tyrosine.

SVD, small vessel disease; HbA1c, glycated hemoglobin; T2DM, type 2 diabetes mellitus.

### 3.3 Additive interaction between phenylalanine and duration of diabetes for SVD

The associations of plasma phenylalanine levels with SVD at different diabetic durations are shown in **Table 4**. In patients with a longer duration of diabetes (≥5 years), low levels of phenylalanine had a statistically significant higher odds ratio for SVD risk in both the univariate and multivariate analyses compared to the patients with a shorter duration of diabetes (<5 years) (OR: 2.36, 95% CI: 1.65, 3.39; OR: 2.05, 95% CI: 1.32, 3.18, respectively). Compared with high phenylalanine (i.e., ≥42μmol/L, the cutoff point of the RCS) and short duration of diabetes (i.e., <5 years, the median), a concurrent presence of both low phenylalanine (<42μmol/L) and long duration of diabetes (≥5 years) markedly increased the risk of SVD [OR: 4.83 (95% CI, 2.97-7.87] compared to low phenylalanine alone (multivariable OR: 1.48, 95% CI: 0.88-2.49) and long duration of diabetes alone (multivariable OR: 2.59, 95% CI: 1.67, 4.02). The additive interaction was significant (RERI: 1.76, 95% CI: 0.05-3.47; AP: 0.37, 95% CI: 0.10-0.63 and S: 1.85, 95% CI: 1.03-3.35) (**Table 5**).

**Table 4:** Odds ratio of phenylalanine levels and different durations of diabetes for the risk of small vessel diseases.

	Duration of diabetes<5 years		Duration of diabetes≥5 years	
	OR (95% CI)	*P*-value	OR (95% CI)	*P*-value
Univariable model				
Phenylalanine, per μmol/L	0.97 (0.95-0.99)	0.002	0.97 (0.96-0.99)	<0.001
<42μmol/L	1.88 (1.16-3.03)	<0.001	2.36 (1.65-3.39)	<0.001
≥42 μmol/L	reference		reference	
Multivariable model 1				
Phenylalanine, per μmol/L	0.97 (0.95-0.99)	0.009	0.97 (0.96-0.99)	<0.001
<42μmol/L	1.75 (1.04-2.95)	0.034	2.34 (1.59-3.45)	<0.001
≥42 μmol/L	reference		reference	
Multivariable model 2				
Phenylalanine, per μmol/L	0.99(0.97-1.02)	0.647	0.98(0.96-0.99)	0.007
<42μmol/L	1.09(0.60-1.98)	0.776	2.05(1.32-3.18)	0.002
≥42 μmol/L	reference		reference	

Model 1 was adjusted for age, gender, body mass index (<18.5, 18.5-23.9, 24-27.9 and ≥28 kg/m^2^), duration of diabetes, smoking, drinking alcohol, systolic blood pressure, high-density lipoprotein cholesterol (<1.0 mmol/L in men or <1.3 mmol/L in women, ≥1 mmol/L in men or ≥1.3 mmol/L in women, unknown), triglyceride (<1.7 and ≥1.7 mmol/L, unknown), antidiabetic drugs, lipid-lowering drugs, and antihypertensive drugs use. Model 2 was adjusted for the variables in model 1 plus tyrosine.

SVD, small vessel disease; HbA1c, glycated hemoglobin; T2DM, type 2 diabetes mellitus.

**Table 5:** Additive interaction of phenylalanine and duration of diabetes for SVD in type 2 diabetes mellitus.

	Univariable model		Multivariable model	
Phe (μmol/L) and dmy(years)	OR (95% CI)	*P*-value	OR	*P*-value
Phe≥42 and dmy<5	reference		reference	
Phe<42 and dmy<5	1.88 (1.16-3.03)	0.010	1.48(0.88-2.49)	0.145
Phe≥42 and dmy≥5	3.03(2.00-4.59)	<0.001	2.59(1.67-4.02)	<0.001
Phe<42 and dmy≥5	7.16 (4.64-11.06)	<0.001	4.83(2.97-7.87)	<0.001
Measure				
RERI	3.26 (0.96 to 5.56)		1.76(0.05-3.47)	
AP	0.46 (0.25 to 0.66)		0.37(0.10-0.63)	
S	2.12 (1.30 to 3.46)		1.85(1.03-3.35)	

The multivariable model was adjusted for age, gender, body mass index (<18.5, 18.5-23.9, 24-27.9 and ≥28 kg/m^2^), duration of diabetes, smoking, drinking alcohol, systolic blood pressure, high-density lipoprotein cholesterol (<1.0 mmol/L in men or <1.3 mmol/L in women, ≥1 mmol/L in men or ≥1.3 mmol/L in women, unknown), triglyceride (<1.7 and ≥1.7 mmol/L, unknown), antidiabetic drugs, lipid-lowering drugs, antihypertensive drugs use, and tyrosine. Significant RERI>0, AP due to interaction>0 or S>1 indicate a significant additive interaction.

AP, attributable proportion; RERI, relative excess risk due to interaction; S, synergy index; Phe, phenylalanine; dmy, duration of diabetes; SVD, small vessel disease.

### 3.4 Potential discriminative values of phenylalanine and duration of diabetes for SVD

The inclusion of phenylalanine and duration of diabetes in the traditional risk factor model significantly increased the area under the curve (AUC) from 0.67 to 0.71 (p<0.05) (data not shown).

## 4 Discussion

In this retrospective study, we found that plasma phenylalanine concentration was negatively associated with the risk of SVD in T2DM. We also detected an additive interaction between phenylalanine and duration of diabetes: the OR of low-level plasma phenylalanine for the risk of SVD was magnified in patients with a duration of diabetes >5 years. Furthermore, phenylalanine and the duration of diabetes significantly increased the discriminative values of the traditional risk factors for SVD in T2DM.

The progression of SVD, i.e., DN and DR, increases the risk of all-cause mortality and blindness (2, 25), posing a significant threat to the quality of life of T2DM patients. Unfortunately, SVD in T2DM develops silently until severe damage has occurred. There is, therefore, a strong need to explore new markers to detect SVD earlier. Our study found that low-level plasma phenylalanine was associated with the risk of SVD in T2DM patients. Similarly, a study based on the GenodiabMar cohort found that phenylalanine was a common risk factor not only for DN but also for both DR and proteinuria (26). In addition, as a downstream product of phenylalanine, low tyrosine also appears to be a marker of microvascular risk in individuals with T2DM independently of other fundamental markers of kidney function (12), which might be relevant to our findings. However, our results were inconsistent with the findings of other studies (27–29), such as that by Zhao et al. which found that phenylalanine modifies insulin receptor beta (IRβ) and inactivates insulin signaling and glucose uptake and was positively correlated with T2DM onset. Previous studies have shown that an association pattern between amino acids and SVD is often influenced by differences in genetics, pathophysiology, culture, and lifestyle among different ethnicities.

In recent years, multiple common pathogenetic mechanisms of DN and DR have been proposed (30, 31), in which microvascular endothelial dysfunction plays a critical role in both kinds of SVD (2). It is well known that nitric oxide (NO) biosynthesis, catalyzed by endothelial nitric oxide synthase (NOS), determines homeostasis of the endothelial structure and function (32, 33). Appropriate levels of NO are able to protect renal endothelial and mesangial cells from apoptosis and fibrosis by inducing the expression of antioxidant genes (34). Tetrahydrobiopterin (BH4) is an important cofactor in the catalytic activity of NOS (35), and its synthesis is mediated by GTP cyclohydrolase-1 (GCH1) (36). When BH4 is insufficient, NOS may become “uncoupled” and produce a large amount of superoxide such reactive oxygen species (ROS), which aggravates oxidative stress and impairs vascular function (37). Furthermore, oxidative stress could cause the disruption of tight junction proteins (TJs), resulting in impaired endothelial barrier function (38).

Phenylalanine is an essential aromatic amino acid obtained through dietary intake in the body and it is mainly used in protein and tyrosine synthesis and, as a substrate, is involved in the synthesis of important neurotransmitters and hormones (39). Our study showed that low-concentration phenylalanine was associated with an increased risk of SVD in T2DM, which may be related to endothelial dysfunction caused by NO synthesis dysfunction which was in turn caused by insufficient phenylalanine. Manasi Nandi et al. found that oral supplementation of L-phenylalanine was capable of enhancing endogenous biosynthesis of BH4 by activating the GCH1-GFRP protein complex, while restoring NO content, reducing ROS levels, and improving vascular endothelial function (14). The role of L-phenylalanine in binding pockets on GCH1-GFRP complexes in the treatment of endothelial dysfunction was also validated in a separate study (40). Furthermore, phenylalanine protects TJs that are located on endothelial cells from damage by inhibiting NF-KB (41). We speculate that disruption of phenylalanine metabolism affects the balance between NO and ROS, thereby affecting endothelial barrier function.

Another potential mechanism was the protective effect of dopamine on vascular endothelium. Peripheral dopamine can directly regulate glucose uptake and lipid metabolism (42), which indirectly reduces endothelial injury caused by hyperglycemia. As a substrate for tyrosine hydroxylase, phenylalanine is indirectly involved in dopamine synthesis and is considered to be a secondary precursor of dopamine (43). Kinya Kuriyama et al. found that dopamine consumption in diabetic rats increased significantly (44), suggesting more substrate may be needed in diabetic patients. Furthermore, dopamine D2-like receptors, especially the D4 receptor, located primarily on the arterial endothelium, have been shown to ameliorate hyperglycemia-induced endothelial dysfunction through the PI3K/eNOS pathway (45). Taken together, we hypothesize that the relative dopamine deficiency in diabetes partially mediates the association between low phenylalanine and a higher risk of small vessel disease.

It is well known that the duration of diabetes is a risk factor for diabetic complications. With the progression of diabetes, oxidative stress and microvascular endothelial dysfunction gradually worsen, eventually triggering vascular-related diseases such as arteriosclerosis and diabetic small vessel disease (46, 47). Our study found that the cutoff value of diabetes duration was 5 years, which could contribute to earlier identification of SVD compared with the 10 years (48) proposed in previous studies. This cut-off point has also been shown to be a risk factor for SVD in multiple studies (49, 50). However, few studies have explored whether there is an interaction between the duration of diabetes and phenylalanine on SVD. In our study, we found a significant additive interaction between the duration of diabetes and low-level phenylalanine on SVD, which indicated that the duration of diabetes and disturbed phenylalanine metabolism may together contribute to the development of SVD in T2DM. However, due to the nature of the retrospective study, we were unable to determine whether SVD caused abnormal amino acid metabolism, which needs to be confirmed in a large cohort study.

Our study has important clinical and public health implications. We found that low-level phenylalanine was associated with an increased risk of SVD, which indicated that SVD, to some extent, can be improved by modulating phenylalanine metabolism. In addition, our study showed an additive interaction between the duration of diabetes (≥5 years) and low-level phenylalanine on SVD, which dramatically widens our appreciation of the etiological mechanisms of diabetic small vessel disease.

This study had some limitations. First, the retrospective design of this study could not verify true causality. Large population-based prospective cohort studies and basic research are required to validate our findings. Second, considering the heterogeneity between Chinese and other ethnicities, caution is required in extending our findings to other populations. Third, there are different degrees of data missing for factors such as LDL-C, HDL-C, TG, and HbA1c in our database. Given that the core of our research was phenylalanine rather than glycolipid metabolism in this study, we regarded the missing values as a category. Fourth, considering that phenylalanine cannot be synthesized by itself and can only be obtained from diet, diet can thus affect phenylalanine levels. Unfortunately, information on diet was not available, thus diet cannot be integrated into the statistical analysis. Finally, the diagnosis of DN and DR in this study only reflected the existing conditions of patients at that time and was not confirmed through long-term monitoring. For example, classification and staging data of DR were not collected, which is one of the limitations of our study.

In summary, our findings detected that low plasma phenylalanine was associated with a high risk of SVD in Chinese patients with T2DM. This association was further amplified in patients with the duration of diabetes ≥5 years. The potential discriminative value of phenylalanine and duration of diabetes for SVD risk was also reflected in the increased area under the receiver operating characteristic curve. Additional prospective cohort studies are required to verify these findings in different ethnic groups with T2DM. Further mechanistic explorations are also needed to reveal the underlying molecular mechanisms of serum phenylalanine in the pathogenesis of SVD.

## Data Availability

The raw data supporting the conclusions of this article will be made available by the authors, without undue reservation.
